# Imitation of coordinated actions: How do children perceive relations between different parts?

**DOI:** 10.1371/journal.pone.0189717

**Published:** 2018-01-03

**Authors:** Sophie J. Milward, Natalie Sebanz

**Affiliations:** Central European University, Department of Cognitive Science, Budapest, Hungary; University of Bologna, ITALY

## Abstract

Young children not only need to learn how to perform isolated actions, but also temporally and spatially coordinated actions such as using a knife and fork. Routes to learning such coordinated actions include imitation and participation in joint action. However, little is known about the mechanisms guiding transmission of coordinated actions through observation and joint action performance. This paper reports an experiment comparing children’s tendency to imitate multiple, coordinated actions following demonstration by a single model acting bimanually (Bimanual Observation condition), two models performing the same actions jointly with one performing each hand action (Joint Observation condition) and a condition in which the child actively takes part in the joint action demonstration by performing one part in coordination with a partner (Joint Action condition). When children were subsequently left alone to perform the task independently, they were more likely to imitate both coordinated actions in the two observation conditions than in the Joint Action condition, with no difference between performance in Bimanual and Joint Observation conditions. It is argued that this is due to children being more able to form a global representation of both actions and the relations between the two when observing from a distance than when actively involved in the task.

## Introduction

During their early years, children develop a repertoire of ever more complex actions and combinations of actions in order to achieve ever more sophisticated goals. Many of these actions involve coordination and thus require children to learn how different parts of the action must be coordinated spatially and temporally. When learning how to catch a ball with two hands, eat with knife and fork, or play a musical instrument, it is not sufficient to know what each hand needs to be doing in isolation. Rather, children need to learn about the relations between different parts of the action. One route to learning about these coordinated actions is by observing other individuals performing them and engaging in imitation.

However, children not only passively watch others, but often engage in joint actions where they perform parts of an action in coordination with a partner performing other parts [1, 2]. A specific case of such active learning is scaffolding [3, 4], which involves an expert, usually a parent or caregiver, guiding a child’s actions so that the child can achieve something that is slightly beyond his/her own capabilities if acting alone. Similarly to riding a bicycle with stabilisers, the experience of performing this action with help then allows the child to perform the action unaided in the future. In the case of both scaffolding during joint action and observational learning from a single model, another individual contributes to the learning process, but the nature of information transfer to the child may be quite different.

Finally, children are also exposed to coordinated actions as they observe others engaging in joint actions. While some joint actions cannot be performed alone, many actions can be performed either alone or together–think of preparing a dish, folding a sheet, or playing the same piano piece either bimanually or jointly with one hand each. The fact that the same actions can be performed by one or more agents raises questions about how children come to imitate coordinated actions that they have observed being performed by a single individual or by two individuals together. Depending on how the joint action is represented, children’s imitation may be focused on only one part, or on both parts and the relation between the two.

The imitation of coordinated actions thus provides a means to investigate how children represent action goals and relations between different parts of actions depending on their own involvement and depending on the number of agents demonstrating the actions. In the current study we investigated whether multiple parts to be carried out concurrently are represented differently by young children when the action is presented to them through demonstration by a single model, when it is demonstrated by two individuals acting together, and when the children participate in a joint action with an adult. In the following, we first review studies that speak to the question of how observation of jointly performed actions may differ from observation of the same actions performed by a single individual. We then review findings on how individuals acting together form joint task representations, to derive predictions for children’s imitation of joint actions that they have engaged in versus that they have observed others engage in.

### Effects of joint action observation on imitation

There is some evidence that from quite early on, children respond differentially to observed actions depending on the number of agents who perform them and the degree of coordination between them. Herrmann, Legare, Harris and Whitehouse [5] showed that children imitated actions more faithfully following demonstration by two models who performed the same action in parallel and in synchrony than following demonstration by a single model performing the action alone, or successive demonstration of the action by two different models. According to their interpretation, observing two agents performing the same action in synchrony enhanced children’s imitation because it created expectations about the conventionality of the observed action. It is not known, however, whether this also extends to the observation of two models performing complementary parts of a joint action rather than performing the same action in parallel.

A study by Fawcett and Liszkowski [6] actually suggests that observing a jointly performed action elicits a tendency in children to replicate the joint action by recruiting a partner even when they could perform the action on their own. They compared imitation following observation of joint actions as well as parallel and individual actions, and found that 18-month-olds were more likely to invite another person to join in the imitated action following observation of a joint action than either parallel or individual actions. Based on these findings, one could speculate that in the absence of a potential joint action partner, children’s tendency to imitate a coordinated action they have observed being performed jointly might be reduced.

Research on automatic imitation of joint action in adults has shown that pairs of participants have a stronger tendency to mimic observed actions when they are performed by two models than when they are performed by a single model, while single individuals showed the opposite pattern. In a first study [7] participants were asked to imitate the tapping finger of a single hand displayed on a monitor. A confederate was also present and responded to a second hand on the monitor, either belonging to the same model (individual action observed) or a different model (joint action observed). Observing joint action triggered a stronger tendency in participants to mimic the actions than observing identical actions performed by a single individual. Converging evidence comes from a more recent study [8] where pairs of participants were asked to tap in synchrony with two hands on a computer screen. The index fingers of the two hands moved in alternation, requiring participants to take turns in tapping. Imitation performance was better when participants observed joint action than when they observed the same actions performed by a single model. These findings can be explained by the assumption that observing joint actions triggers corresponding joint action plans in observers, spanning both participants’ actions. Rather than specifying only one part, for example the part that they are supposed to imitate, observers treat all observed actions as a single unit, which consequently primes joint imitation and processing of multiple actors as the unit to be imitated.

### Joint task representations in joint action

Research on action planning indicates that very similar motor planning processes are involved in bimanually coordinated actions and in equivalent joint actions. In an EEG study by Kourtis et al. [9] participants received cues either instructing them to prepare for lifting two glasses and clinking them together by themselves, or cues instructing them to lift one glass and to clink it together with a glass moved by a task partner. The CNV, a marker of motor preparation, was equally pronounced in the two conditions, indicating that participants prepared for the two actions regardless of whether they were going to perform both of them or only one of them. In contrast, performing one part of the action in the absence of a partner resulted in less motor preparation. This indicates that in the bimanual and in the joint condition, participants’ planning was guided by a task representation that specified both actions to be performed and the relation between them.

If participating in joint actions leads to task representations that specify the two different actions and the relations between them, then this may facilitate learning transfer to situations where individuals perform the whole task by themselves, as the same relations need to be instantiated by the individual. However, when people do not have a shared goal, there is much evidence to suggest that they experience interference between their own and another’s task. The tendency to form representations of co-actor’s tasks [10] can result in detrimental interference effects due to a lack of a clear separation between self and other [11]. Developmental work has shown interference effects in children [12, 13] and has highlighted the role of Theory of Mind in keeping self and other apart. Milward, Kita & Apperly [14] found that children who were better at avoiding interference from a co-actor’s task were also better on a battery of Theory of Mind tasks. The authors argue that this is because children who are better at Theory of Mind are better able to keep representations of self and other separate. If children do not perceive their part and another’s part as relating to a shared goal during joint action performance, they might experience interference between the parts or simply focus on their own part. This, in turn, would reduce their tendency to later reproduce both parts when acting alone.

### The present study

This study aimed to test to what extent active participation in joint action and joint action observation allow for a global representation of both parts of a coordinated action and the relations between them. As our review of related studies shows, there is some reason to predict that participating in joint action may lead to task representations that can be transferred to individual performance. However, limitations to imitation of joint action are expected to the extent that children fail to perceive the shared goal binding different parts of a joint action together. Observation of joint actions may also lead to limited individual imitation if children fail to perceive the relation between the different parts and represent them as separate actions or roles to be performed by different agents. Alternatively, it could be that observation of joint actions leads to encoding of an overall, joint goal, requiring multiple actions but not including agent information. The latter would result in equal imitative performance following observation of either a joint action or an individual, bimanual action.

An age range of 2.5–6 year-olds was chosen because this is a period during which both Theory of Mind and joint action representations show significant maturation. It has been argued that children from around 2 years already have the ability to form joint goal representations [15]. Explicit Theory of Mind has been argued to develop around 3–4 years [16], with an implicit level of mind-reading being apparent from early infancy [17]. This allows for informative variance across the age range tested here.

**Fig 1:**
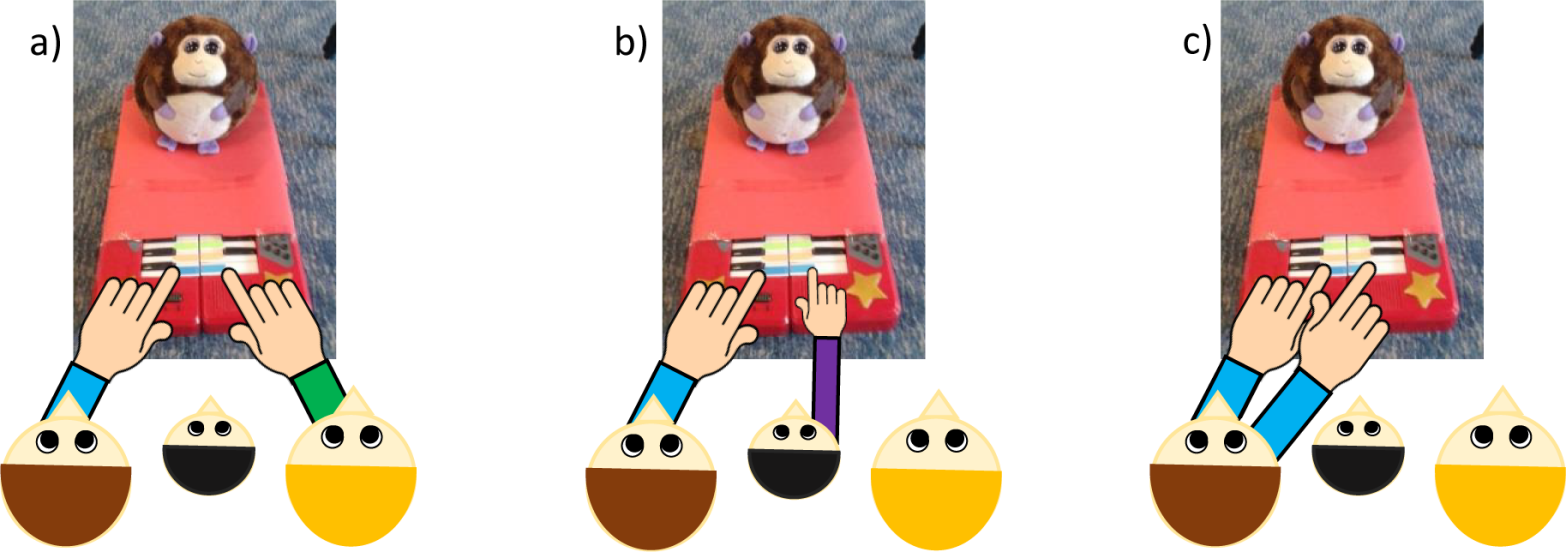
Illustration of the three demonstration types. a) Joint Observation. Experimenter and Confederate demonstrate tune in synchrony; b) Joint Action. Experimenter plays one part of the tune and scaffolds the child playing the other part in synchrony; c) Bimanual Observation. (Baseline) Experimenter demonstrates tune bimanually.

A musical task was designed that involved playing two sequences of 5 key-presses on adjacent keyboards in synchrony, so that two keys were always pressed at the same time, producing pairs of harmonious tones. The two synchronous sequences could either be performed by a single actor performing both sequences bimanually or by two co-actors each performing a single sequence unimanually (see Fig 1). Children witnessed demonstrations of the task either by observing a single person acting bimanually (Bimanual Observation condition), observing two people acting unimanually in synchrony (Joint Observation condition), or by actively taking part in the task by performing one part while a co-actor performed the other part in synchrony (Joint Action condition).

By first demonstrating a task featuring two synchronous parts that could be performed by one or two individuals, we could test whether children represent both parts globally or each part separately (or even just one part alone) by measuring their behaviour on a subsequent imitation task. Global representation should make it easier for children to imitate both parts and the relationship (temporal synchrony) between the two. Separate representations of each part should be more effortful to keep in mind and should therefore be more likely to lead to imitation of only one part.

## Method

### Participants

Participants were 151 2.5–6 year-olds, of which 26 were excluded for the following reasons: Misuse of apparatus (e.g. hitting all buttons with the fist, attempting to break apparatus, n = 10); Failing to make any response (n = 8); Intervention by a parent (n = 4); Refusal to take part (n = 3): Technical error (n = 1). This left a remaining 125 participants with a mean age of 58.92 months ranging from 30–82 months (Joint Observation: *n* = 45, Mean Age = 59.31, SD = 13.00; Joint Action: *n* = 43, Mean Age = 58.40, SD = 13.72; Bimanual Observation: *n* = 37, Mean Age = 58.81, SD = 13.81). All participants were sampled from Birmingham’s Think Tank Science Museum in the United Kingdom, where families were recruited beforehand through advertisements on parenting websites and were offered free entry to the museum in return for taking part. This study was approved by the United Ethical Review Committee for Research in Psychology (EPKEB). Written consent was obtained from caregivers of all children who participated. Children all received a sticker as a reward.

### Stimuli

Stimuli consisted of a ‘Music box’ toy, made from two toy keyboards (48x12x2.5cm) placed together so the keys from each keyboard were facing inward and touching (see Fig 1). Only the first three keys of each keyboard were required for the task, so the remainder were covered with red card, on which was placed a stuffed monkey toy (20x20x20cm), also displayed in Fig 1. The six visible keys were coloured using blue, orange and green stickers, so that keys from the right- and left-hand keyboards that were directly touching were coloured the same. A Canon HD20 video camera was used to record performance.

### Design and procedure

Children were tested on the floor in a quiet classroom in the museum. The experiment task lasted around 2–3 minutes and consisted of two trials, both of which were video-recorded. One of three confederates (one male and two female) was employed in the experiment. The confederates could not be kept the same throughout the experiment due to circumstances beyond the authors’ control (the initial confederate was unable to continue until completion of data collection). The proportion of children tested by a male or female confederate in each level of the dependent variable of interest, Demonstration Type, was equal (tested by male confederate: Joint Observation: 55.6%; Joint Action: 51.2%; Bimanual Observation: 59.5%) with equal numbers of male and female participants tested with each confederate gender in each condition. However, the mean age of participants tested by male and female confederates was not equal (Male confederate: Mean age = 66.30 months, SD = 10.87, Range = 30–82 months; Female confederate: Mean age = 49.66 months, SD = 10.12, Range = 36–80 months). This issue is taken into consideration in the analyses. The female experimenter (SJM) remained constant.

The experimenter showed the child the music box and asked him/her to sit down in the centre. She then sat on the child’s left and the confederate sat on the child’s right. The experimenter then explained, “What we’re going to do is to play a game using this music box. See the box here [points to box] has lots of different buttons in different colours, and each makes a different sound. And this is Monkey [points to Monkey]. There’s a tune that Monkey really likes, so we’re going to play the tune for Monkey using the buttons. First we’ll play it together, then it will be your turn to play the tune on your own. Does that sound ok? [Wait for affirmative response from child]. Ok, first I’ll show you what to do. First we press the blue button, then the orange button, then the green button, then the orange button and then the blue button. The following part of the task varied depending on the condition (see Fig 1).

#### Joint observation condition

[Addressing confederate] *Confederate’s name*, can you do that with me now?” The confederate and the experimenter then followed the sequence given in the instructions, with the experimenter pressing buttons on the left and the confederate pressing buttons on the right each with a single finger and in synchrony with one another at a comfortable tempo (approximately one tone per second).

#### Joint action condition

This was identical to the Joint Observation condition, except that the experimenter addressed the child rather than the confederate in asking him/her to play together. The experimenter modulated her responses so that they occurred in synchrony with the child’s, by placing her finger over the correct button but waiting to press it until the child had placed his/her finger over his/her correct button. If the child placed a finger over the incorrect button, the experimenter verbally guided him/her to the correct response (e.g. “now the green button…”).

#### Bimanual observation condition

This was the baseline condition. It was identical to the Joint Observation condition except that rather than instructing another person to play along with her, she said “Shall I do that now?” She then played both left and right buttons herself, using her left and right hands respectively, at a comfortable tempo (approximately one tone per second).

The demonstration in all conditions produced the same outcome, which was a sequence of harmonies between the two notes produced by each of the buttons (each one a typical keyboard note). The two keyboards were spatially arranged (see Fig 1) so that pressing two buttons of the same colour always resulted in two different but harmonious tones. This implies that in all conditions the tune was comprised of two melodies to be played in synchrony. The sequence was always repeated three times, with the experimenter verbally acknowledging the repetition each time (e.g. “and again…”, “one more time…”) to indicate that each was a discrete sequence. The experimenter then addressed the child, “Do you think you can do that on your own now? I have to go over here for a moment, but while I’m gone can you play the tune for Monkey?” The experimenter and confederate then both left and moved to separate parts of the classroom and out of the child’s view if s/he was looking at the apparatus. They then waited for 20 seconds. If a participant failed to make any response within the first 5 seconds, the experimenter gave a prompt, “Can you play the tune for Monkey?” After 20 seconds, the experimenter and confederate returned their seating positions next to the child to begin the second trial. The experimenter said, ‘Well done! Shall we try another one? There’s another tune that Monkey really likes. First we’ll do it together and then it will be your turn to do it on your own. This time, first we press the green button, then the orange button, then the blue button, then the orange button, then the green button. *Confederate’s name* can you do that with me now? The rest of the procedure was identical to the first trial. On completion, the experimenter gave the child positive feedback and a sticker.

### Video coding

All trials were video recorded and rated on several measures by a single coder. A subset of 25% of these recordings chosen at random were coded by a second, independent coder who was trained on the coding scheme and kept ignorant of the hypotheses of the experiment. Two aspects of children’s behavior were of interest: a) whether they responded uni-manually or bi-manually, and b) how accurate their responses were. Accuracy was measured by counting the number of correct transitions between two keys within a sequence. For example, on Trial 1 transitions from blue to orange, orange to green, green to orange and orange to blue were counted as correct. Additionally, if children repeated the sequence of 5 presses more than once, then it would be necessary to press the blue button twice in a row, once for the last press of the first sequence (i.e. [blue, orange, green, orange, **blue**], and then again for the first press of the following sequence [**blue**, orange, green etc.]). For Trial 2, the same was true for green presses, which occurred both at the start and the end of each correct sequence. Therefore, two blue presses in a row (on Trial 1) or two green presses in a row (on Trial 2) were also marked as correct. These episodes of repetition of the start/end colour button in a sequence were not counted as correct if they appeared on the first transition of a trial, as this could not have been a combination of the end of one sequence and the start of another. Additionally, if more than two start/end-colour button presses occurred in a row, only one transition was counted as correct.

## Results

The key dependent measure was the type of action carried out by the child on their first key press, given that this is the purest measure of representation of the preceding demonstration without any additional influence of the child’s own experience with the apparatus. This was divided into two main response types: ‘One Key’ responses, which involved use of a single finger to press a single key, and ‘Two Key’ responses, which involved pressing down two keys of the same colour in synchrony. Although the specific action used to produce Two Key responses varied (using a single finger from each hand to press buttons bimanually, using two fingers from the same hand or using a single finger from a single hand placed in the centre of the two keys), responses were divided dichotomously in this way in accordance with the original hypothesis that type of demonstration of the tune would modulate representation of one part of the task (one key) or both (two keys).

**Fig 2:**
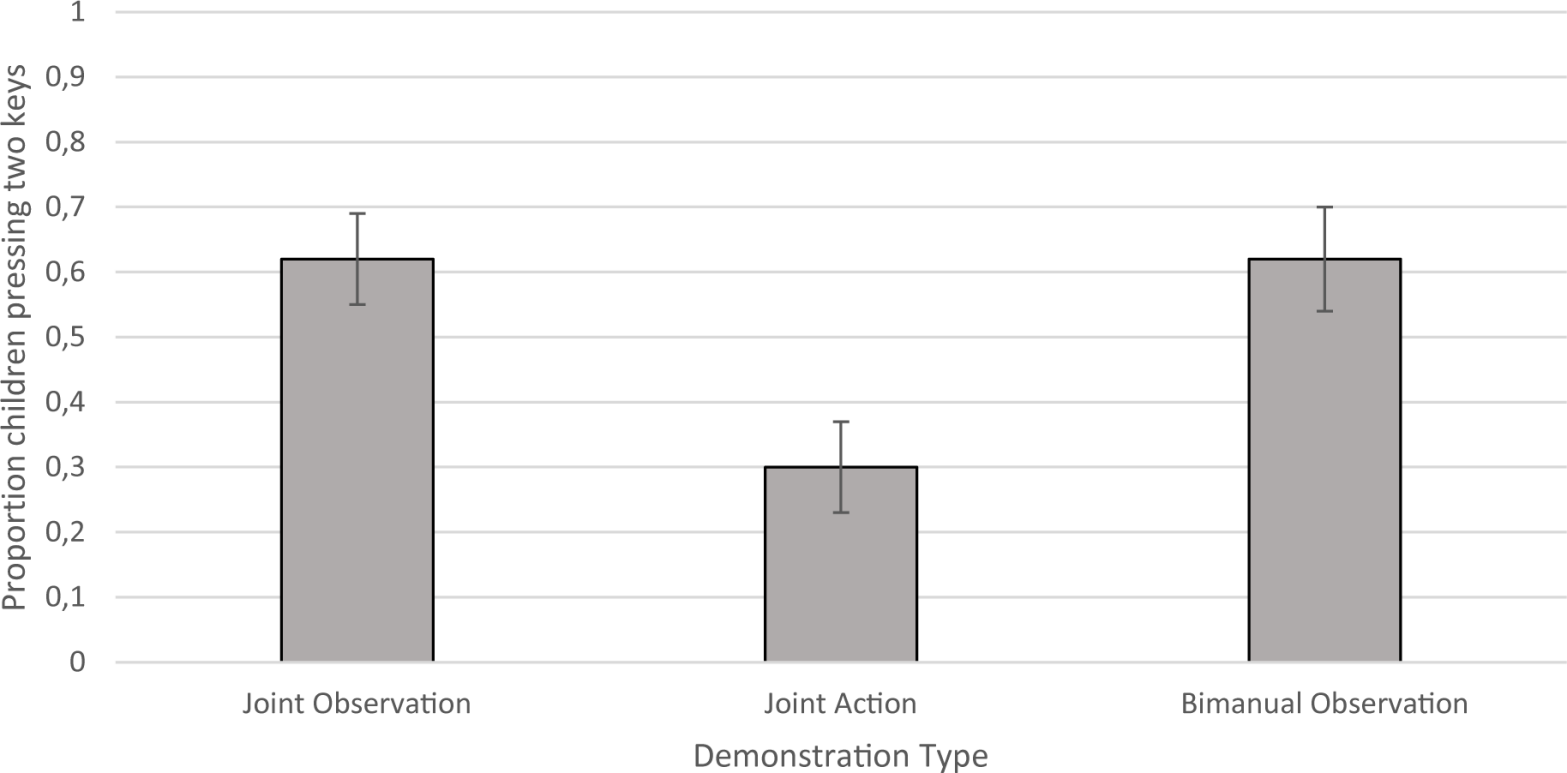
Proportion of children pressing two keys in synchrony on their first key press on Trial 1 for each Demonstration Type.

Fig 2 shows the proportion of children who made a Two Key response on their first action towards the apparatus. A logistic regression with Demonstration Type, Age in months and Confederate Gender as predictors and First Response Type as the dependent variable (with Bimanual Observation as the reference category) revealed a significant overall model (*χ2* = 27.73, *p* < .001) explaining 26.5% of the variance associated with first response type on the task and correctly classifying 65.6% of cases. Participants in the Joint Action condition were less likely to make a Two Key response with their first action than those in the baseline Bimanual Observation condition (*β* = -1.44, *SE β* = .51, *p =* .005) but there was no difference between the Joint Observation and Bimanual Observation conditions (*β* = -.01, *SE β* = .49, *p =* .98). The same analysis was repeated with Joint Observation as the reference category, demonstrating a difference between Joint Observation and Joint Action conditions (*β* = -1.44, *SE β* = .51, *p =* .005). There was also a trend for older children to make bimanual responses (*β* = .04, *SE β* = .02, *p =* .052). There was no effect of Confederate Gender (*β* = .74, *SE β* = .50, *p =* .14).

**Fig 3:**
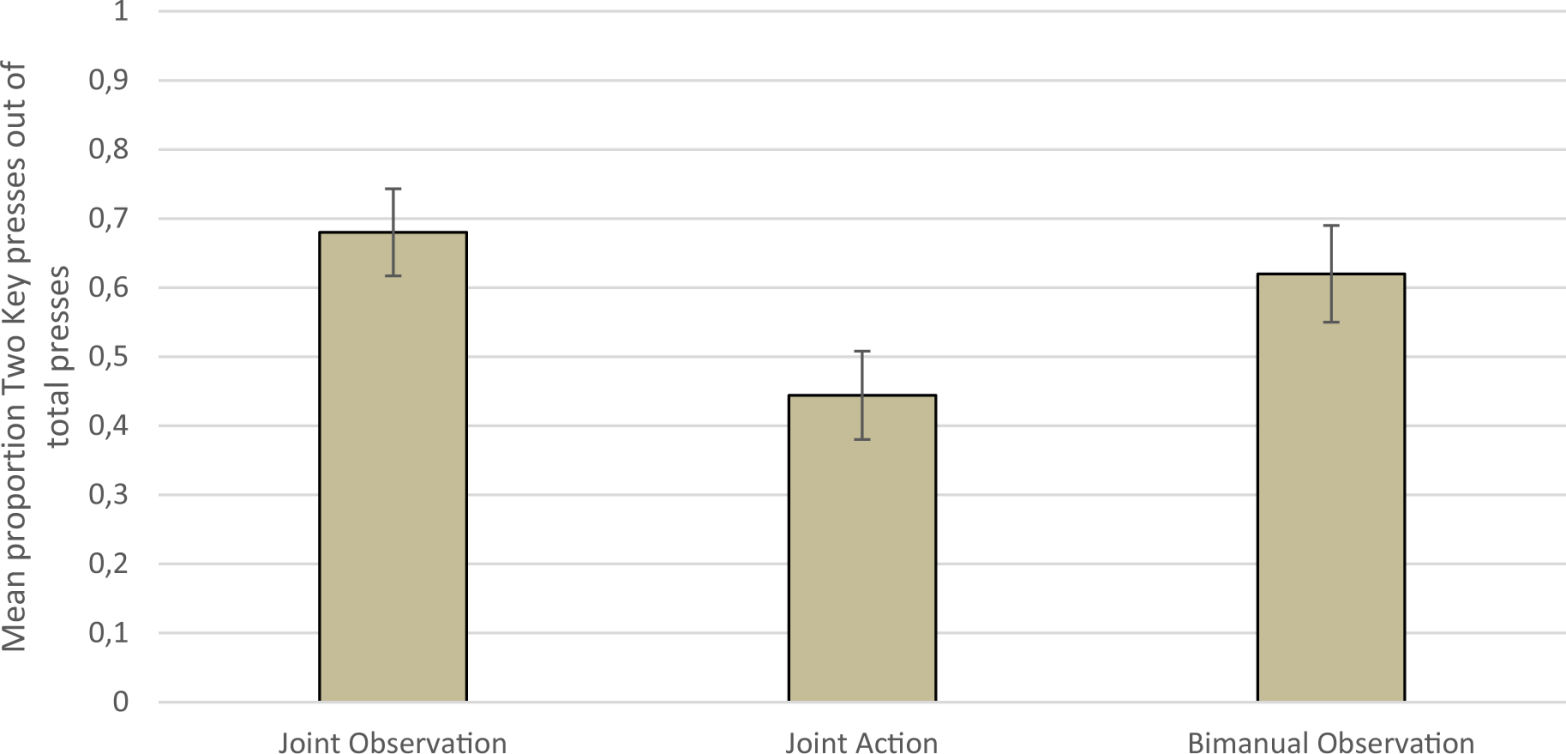
Mean proportion of Two Key presses out of total presses for each Demonstration Type.

Data were then investigated in terms of overall performance over the entire experiment (all button presses made during Trial 1 and Trial 2). Firstly, there was no difference in total number of presses made in each Demonstration Type (Mean = 44.42, Standard Deviation = 21.80) as demonstrated by a one-way ANOVA (*F*(2,117) = 2.20, p = .12). Proportion of Two Key presses out of total key presses was calculated for each participant and entered as the dependent variable in a univariate ANOVA with Demonstration Type as the independent variable and Age in months and Confederate Gender as covariates. These covariates were included to be certain that any effect of Demonstration is not a result of age effects that are somewhat confounded with confederate gender. This showed a significant effect of Demonstration Type (*F*(2, 117) = 3.60, *p* = .03, *η*^*2*^ = .06) after controlling for Age in months (*F*(1, 117) = 3.47, *p* = .07, *η*^*2*^ = .03) and Confederate Gender (*F*(1, 117) = 7.63, *p* < .01, *η*^*2*^ = .06). Pairwise comparisons (see Fig 3) showed that children were more likely to produce a higher proportion of Two Key presses in the Joint Observation condition than in the Joint Action condition (Mean difference = .24, *p* = .01) and there was a nonsignificant trend for higher proportion of Two Key presses in the Bimanual Observation baseline than in the Joint Action condition (Mean difference = .18, *p* = .07). There was no difference between the two Observation conditions (Mean difference = .06, *p* = .54). The same pattern of results was found when each trial was entered into a separate ANOVA (Significant effect of Demonstration Type for Trial 1: *F*(2, 119) = 4.23, *p* < .02, *η*^*2*^ = .07; and Trial 2: *F*(2, 118) = 3.30, *p* = .04, *η*^*2*^ = .05).

Finally, accuracy of responses was investigated. The number of correct transitions between keys in the target sequence (as outlined in the Methods) were analysed. For Two Key responses, the simultaneous pressing of two buttons was counted as a single press and thus the transition to the next press (whether One Key or Two Key) was counted as a single transition. Correct presses were then calculated as a proportion of the total transitions made for each type of response. A univariate ANOVA with mean proportion of correct transitions out of total transitions (Joint Observation = .81; Joint Action = .81; Bimanual Observation = .77) as the dependent variable, Task Type as the independent variable and Age in months and Confederate Gender as covariates found no effect of Task Type (*F*(2, 116) = 1.33, *p* = .27, *η*^*2*^ = .02) after controlling for Age in months (*F*(1, 116) = 19.87, *p* < .001, *η*^*2*^ = .15) and Confederate Gender (*F*(1, 116) = 2.52, *p* = .12, *η*^*2*^ = .02).

For all measures, a second rater coded a random 25% of the videos (n = 32). For type of response on the first Trial, raters were in 100% agreement (Cohen’s kappa = 1). For proportion measures, first and second raters’ scores were significantly correlated with each other (Proportion Two key presses: *r*^*2*^ = .92, *p* < .001; Proportion correct transitions: *r*^*2*^ = .79, *p* < .001).

## Discussion

These data show that children are more likely to imitate two synchronous and complementary parts of an action following observation of those parts being performed by others than following active participation in which they perform one of the actions themselves with a co-actor. This suggests that children at this age interpret multiple coordinated actions as belonging to a global task goal when observing others perform them, but do not do so when they are actively involved in a joint task. The fact that there was no difference between the two observation conditions provides support for the conclusion that observation of joint action involves agent-neutral representation of task contributions, because if agent information was included in task representations then observing two agents should be very different from observing a single agent.

Contrary to the current results, previous research has suggested that there are differences in representation of multiple versus single agents following observation [5, 6, 7, 8]. However, in Herrmann et al. [5], actors were performing two identical actions in parallel, each directed towards a separate object. Thus, the synchronicity of the actions was not purposeful in a physical sense, and could therefore be interpreted as providing information about social convention rather than achievement of a joint goal. The current study also showed actors performing identical actions in synchrony, but in this case the purpose of the synchrony was to achieve a concrete goal (producing harmonious tones making up a specific melody) by acting on different parts of a shared apparatus. The difference between these two cases may be the additional information about the relationship between actions and/or agents that are observed. In a joint task, the interactions between the actors, their actions and their goals are intrinsic to the task, whereas in parallel tasks these interactions are unlikely to be relevant or even present.

The difference in findings from Fawcett & Liskowski [6], where children tried to recruit a partner for joint action following joint action observation, can be explained by the pragmatic set-up of the two studies. In the current study, the Experimenter did not make it obvious that she could be recruited during the test phase, as she made an excuse about having to leave and then sat at some distance behind the child. Thus, both studies provide evidence that observation of joint actions results in representation of both parts of the task and children subsequently attempt to reproduce both. However, children’s recruitment of a partner in the former study may have been the result of an additional normative expectation, due to the way the task was framed, that tasks observed being performed jointly should subsequently be imitated jointly. This suggests some awareness of the number of agents in a task even in observational contexts, but at a more explicit level than the task representations addressed in the current study. The fact that when children are required to replicate the actions independently, they are as able to do so as when they observe a single model suggests that the underlying representation of the parts of the task and their relations is the same in joint and individual contexts.

The relatively low occurrence of imitation of both parts of the task in the Joint Action condition suggests that children did not represent a global goal involving all the actions they had witnessed during the demonstration, but rather represented them as separate actions with separate goals. The nature of the instructions in the current task, which specify an ambiguous goal of “playing the tune”, may, in fact, have induced children to infer an individual goal to play the unimanual sequence, rather than a joint goal to produce both sequences in harmony. This is quite possible, considering the common occurrence of scaffolding in children’s learning experiences at this age. In other words, children may have interpreted the partner’s actions in the learning phase as pertaining to a different goal (teaching) than the child’s own actions (learning). For this reason, they then interpreted the instruction to play “the tune” as referring to their sequence alone. If this is the case, the increased imitation in the Joint Observation condition would still suggest inference of a joint goal shared between the two observed actors. It would be interesting to identify which specific factors in active versus observational contexts induce individual or joint goal construal.

The current study alone cannot distinguish between children representing only their own part and an individual goal in the Joint Action condition versus representing their own part and goal, but also their partner’s. Existing evidence that children from at least 4 years old do co-represent a partner’s task apparently automatically [12, 13, 14] and that they show increased motor system activation when actively engaged in the task than when observing [18] point towards the latter argument, but this should be investigated further.

The reduced performance in the Joint Action condition may seem to contradict existing findings that have shown positive effects of active experience on a task with subsequent understanding of the goal of the task, even in infancy [19, 20, 21]. However, the current study differs in that a) performance is measured in imitative tendency rather than goal understanding, and b) experience is divided between two actors, so the child does not experience all parts of the task. It may well be that active experience does help infants and children to understand the goal of a task, but this does not mean that it helps them to learn the means for achieving those goals, especially if they do not have experience with all parts of the task. Additionally, we know little about how active experience might aid understanding of joint goals. It would be very interesting, for example, to see whether experience in the Joint Action condition of the current task gave children an insight into the joint goal of another pair carrying out the same task.

One potential shortcoming in the current study is that children in the Joint Action condition only got experience with one part of the task. This could be problematic if children then found it difficult to inhibit doing exactly the same as what they did in the Demonstration Phase on the Test Phase. This cannot be ruled out from our results. However, the fact that there is no difference between the two observation conditions lends credence to our explanation of agent-neutral representation as being the cause of replication of multiple parts of this task. It should also be noted that even though experience is only with one part of the task in the Joint Action condition, this is still more than in either of the observation conditions, in which children do not have any experience of either part of the task. If beneficial to learning, as has been suggested, one should expect that experience of one part of the task would free up cognitive resources which could be focused on the unpractised part.

The lack of any effects of Demonstration Type on accuracy fits with the literature on perception-action links suggesting a bidirectional association between perception and action that can aid learning either from perception to action [22] or from action to perception [20]. Accordingly, it seems that the effectiveness of active learning and observational learning may be comparable in controlled circumstances, at least in the case of learning a single part of a task.

Taken together, our findings shed light on the mechanisms that are involved in processing multiple parts of a joint action, and how these differ depending on the level of engagement an individual has with the interaction. Here it is argued that passive observation of a task being carried out by others allows children to form a representation of the joint action goal with agent-neutral representation of the different parts involved, which makes it easier for the observer to then combine and replicate these parts him-/herself. In contrast, active participation in a joint action results in agent-specific representation of one’s own (and perhaps one’s partner’s roles and goals) in the task, which is then more difficult to combine and replicate as an individual. These differences highlight an important methodological concern when studying social cognition, which is that the observational or participatory context of the subject may result in different processing of information [23]. The findings may additionally contribute to more applied research on teaching methods, providing evidence that when tasks involve tightly coordinated actions, passive observation may be more beneficial than scaffolded, active experience.
